# Sailfish migrations connect productive coastal areas in the West Atlantic Ocean

**DOI:** 10.1038/srep38163

**Published:** 2016-12-01

**Authors:** Chi Hin Lam, Benjamin Galuardi, Anthony Mendillo, Emily Chandler, Molly E. Lutcavage

**Affiliations:** 1 Large Pelagics Research Center, School for the Environment, University of Massachusetts Boston, P.O. Box 3188, Gloucester, MA 01931, USA; 2 School of Marine Science and Technology, University of Massachusetts Dartmouth, Fairhaven, MA 02719, USA; 3 Keen M International Fishing Charters, Isla Mujeres, Quintana Roo, Mexico

## Abstract

Isla Mujeres, Mexico is home to one of the most well-known aggregations of sailfish. Despite its fisheries prominence, little is known about this sailfish assemblage, or its relationship to other aggregation sites in the western Atlantic. In January 2012, April 2013 and 2014, we deployed 34 popup satellite archival tags on sailfish in order to study their behavior, population connectivity and biophysical interactions. Sailfish were monitored for up to one year, and displayed (1) predominantly shelf associated activity (2) occupancy of the Yucatán Current near Isla Mujeres for up to five months and (3) subsequent dispersals from the Yucatán to productive coastal areas in the Gulf of Mexico, the Caribbean Sea and along the South American coast. Tagged sailfish occupied a median temperature of 26.4°C (interquartile range, IQR = 2.5 °C; range = 12.3–33.3 °C) and median depth of 4.4 m (IQR = 19 m; range = 0–452 m). Diel activity was present and individuals made distinctive descents before sunrise and sunset. Tracking missions of sufficient duration (~1 year) revealed previously undetected connectivity between western Atlantic sailfish fisheries and pelagic longline catches, and highlighted how fishery independent tagging can improve understanding of sailfish migrations and behavior for assessment and management.

Sailfish (*Istiophorus platypterus*), an epipelagic billfish, are prized by recreational fishermen^1^,^2^,^3^,^4^, and provide major nutritional, economic and cultural benefits for artisanal fishing communities^5^,^6^,^7^,^8^. Both eastern and western stocks are considered likely overfished, with biomass below, and fishing mortality above, maximum sustainable yield^9^, although uncertainties are recognized in the stock assessment conducted by the International Commission for the Conservation of Atlantic Tunas (ICCAT)^10^. There are no ICCAT harvest controls for sailfish, but countries such as the United States and Venezuela have domestic regulations that prohibit sale^11^, designate gear modifications^12^,^13^ and/or stipulate area closures^14^,^15^. Mandatory live release from circle hooks has also been proposed to reduce bycatch mortality^16^, but is shown to be insufficient to rebuild the western stock^17^. To improve sailfish population status, basic understanding of movements, behavior and the extent of dispersal are needed to determine stock structure^18^, identify reproductive strategies and estimate productivity^10^.

Conventional and electronic tagging characterize movements of Atlantic sailfish as more restrictive than other billfishes, and localized to coastal areas^19^,^20^,^21^,^22^,^23^. However, the short deployments (median duration = 11; maximum duration = 145 days) for which electronic tags have remained on sailfish to date have limited our understanding of broad-scale spatiotemporal information on their dispersal and behavior. No trans-Atlantic movement has been documented by any tagged fish, which provides little support to the evidence of genetic homogeneity in sailfish across the Atlantic^24^,^25^.

Despite reproduction being a key driver for migration^26^, major gaps in its characteristics for sailfish are contributing to stock assessment uncertainties. Sexual maturity (L_50_) is reached at 135–180 cm lower jaw fork length (LJFL), depending on location^27^,^28^,^29^, and multiple spawning episodes may occur over a long (>6 months) reproductive season^27^,^30^. Reproductively active sailfish are highly transient, and spawn in multiple sites and at different times throughout the western Atlantic^31^ as documented by larval collections off the South Atlantic Bight^32^, Southeast Florida^33^, Bahamas^34^,^35^, Straits of Florida^32^,^36^, and northern Gulf of Mexico^37^,^38^,^39^. Eggs were collected off Belize^40^, while spawning condition fish were found off southeastern Brazil from October to March^41^,^42^ and along the northern coast of South America year-round^27^. Surprisingly, movements of sailfish connecting these various spawning areas are still not characterized.

The seas off Yucatán Peninsula, Mexico are a highly productive^43^ aggregation area for seabirds, sharks, billfishes, marine mammals and their prey species, where local upwelling^44^,^45^ provides favorable foraging conditions for the diverse marine assemblage. In particular, the island of Isla Mujeres is recognized as one of the world’s top “hotspots” for recreational fishing for billfishes. While peak availability of sailfish for recreational fishing occurs from January through late April, little is known about (1) the extent of their residency in the area, (2) their subsequent migrations in summer and fall, and (3) their relationships to the pelagic longline fishery, operating on distant high seas. To shed light on these issues, we present the first long-term (up to one year) records of dispersal, depth, temperature and oceanographic associations of sailfish collected via electronic tags over three field seasons off Isla Mujeres, Mexico.

## Results

Of the 34 popup satellite archival tags or PSATs deployed, seven (21%) failed to report, and seventeen (50%) were shed prematurely and transmitted data (Table 1). X-Tag reporting rates for the three release years are: 75% (n = 8), 63% (n = 11) and 90% (n = 10). Of the nine reported at term, one MiniPAT reported at four months, three X-Tags at six months, and five X-Tags at a full year, one of which was carried by the smallest tagged sailfish (137 cm LJFL) in this study. These represent the longest deployments of PSATs on sailfish to date. One MiniPAT and three X-Tags reported a popoff position but did not transmit or return additional data. The cause for transmission failure is unknown, except for Tag 2012–20562. This tag popped off on schedule after one year, reported its first positions off Cozumel, and was subsequently washed ashore, likely with its antenna pointing sideways or buried, ending transmission. Tag 2013–20560 was recovered off the fish before the programmed release date by our crew while fishing in the tagging area, and Tag 2012–20557 by colleagues off Cape Eleuthera, Bahamas shortly after the tag detached as scheduled. Non-reporting Tag 2012–20565 was returned after a landlord found it in a vacated apartment in central Louisiana. Reporting Tag 2012–20027 was located with Google Earth™ on land near a private marina in Tuxpan, Mexico, presumably recaptured on the sailfish, but could not be recovered.

Tagged fish remained at liberty from 1 to 365 days (mean ± SD or 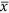 = 174 ± 137, n = 28). Sensor data documented evidence of predation (e.g., complete darkness at daytime) for three sailfish: one just one day post-release and two after >1 month post-release. Predation-associated impacts did not damage the tag or antenna, and thus made the data available for deducing the plausible fate of the tagged individuals.

### Horizontal movements

**Figure 1:**
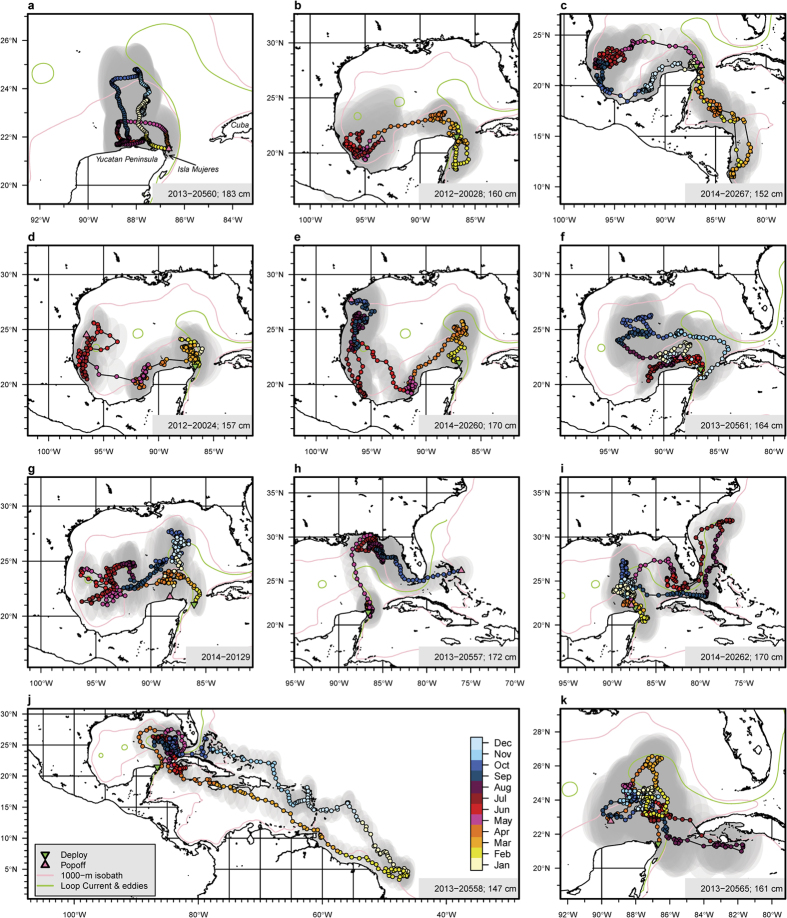
Representative tracks of sailfish. Positions are color-coded by months. Confidence regions at 95% associated with estimated positions are indicated by grey shading. Tag IDs and fish lower jaw fork lengths are noted in the bottom right labels. Tagging locations, green triangle; popoff locations, red triangle; contour of bottom depth at 1000 m, pink line; Loop Current and eddies (annual average), green lines. Refer to Fig. S2 for names of geographic and oceanographic features. Maps were generated in R 79 (v2.15.2).

Tagged sailfish dispersed over a wide geographical range between 3–30°N and 45–97°W, connecting productive coastal areas throughout the Gulf of Mexico (GOM), the Caribbean Sea, and along the South American coast (Fig. 1 & Fig. S1). Four sailfish returned to the Yucatán area, within 60 km of where they were tagged after >300 days at liberty and having traveled as far as 1548 km (straight-line distance) away from the tagging location (Table 1). After release, sailfish remained on the continental shelf off Cancún or on the Campeche Bank (Fig. 1a) between 4 and 147 days (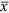 = 57 ± 40, n = 18; Table 1). Sailfish then dispersed south to Belize (Fig. 1b) and to Costa Rica (Fig. 1c). Others moved west in the Bay of Campeche towards the East Mexico Shelf (Fig. 1b,d and e) and western GOM (Fig. 1f,g). They also headed north along the Loop Current into the Mexico Basin, reaching the Mississippi-Alabama Shelf (Fig. 1h) and West Florida Shelf (Fig. 1h–j), and through the Straits of Florida. From the Bahamas, sailfish moved either north to the Blake Plateau (Fig. 1i), or south into the Caribbean Sea, and as far as the NE Brazilian coast (Fig. 1j). On return journeys to the Yucatán Peninsula, sailfish spent time near Jamaica and Cuba (Fig. 1f,j and k), as well as locations in GOM (Fig. 1c,f,g and i). Traveling between coastal destinations, sailfish movements in offshore, pelagic waters were rapid and directed.

**Figure 2:**
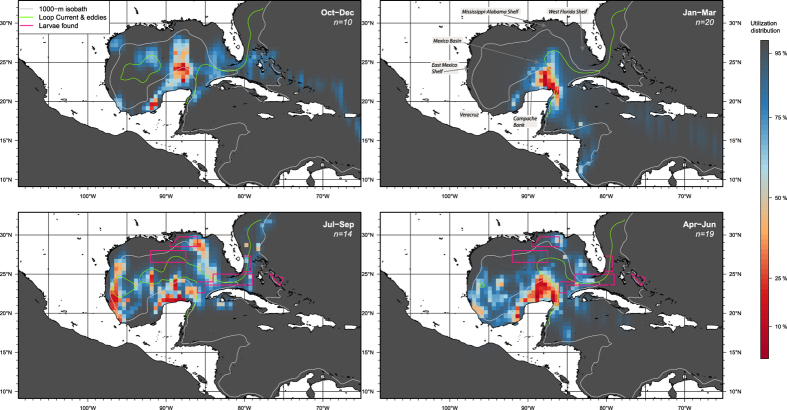
Seasonal spatial use by tagged sailfish. Utilization distribution is plotted in false color on a 0.5° grid. Spawning occurs between April and September in areas defined by magenta borders. Contour of bottom depth at 1000 m, pink line; Loop Current and eddies (seasonal average), green lines. Maps were generated in R 79 (v2.15.2).

**Figure 3:**
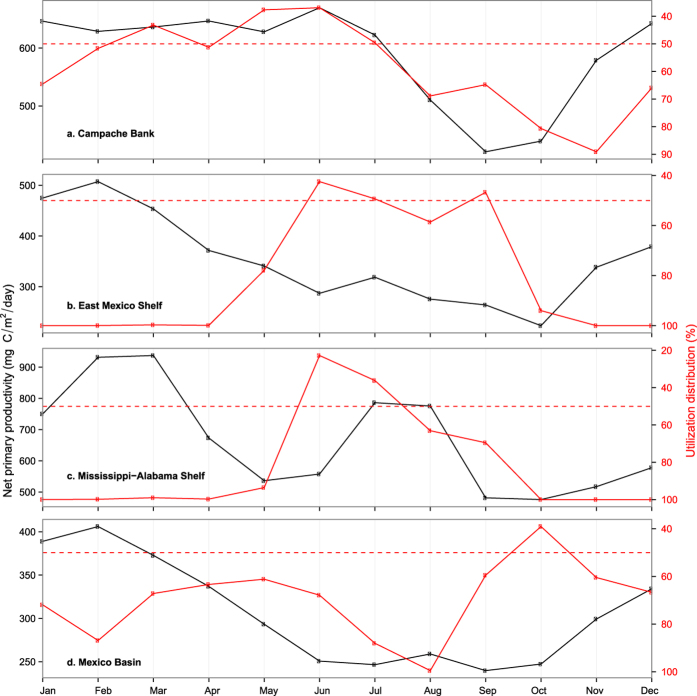
Time series of net primary productivity and sailfish utilization distribution. Monthly averages of net primary productivity (2012–2015) and sailfish utilization distribution (UD) on the Campeche Bank (**a**), East Mexico Shelf (**b**), Mississippi-Alabama Shelf (**c**), and in Mexico Basin (**d**). These areas are selected as utilization hotspots with their boundaries defined by the quarterly 50% UD contours (c.f. Fig. 8). The smaller the UD, the higher utilization an area gets. Notice y-axes are scaled for better visualization.

Utilization hotspots, defined by ≤50% utilization distribution, were found at multiple sites in the Gulf of Mexico (Fig. 2). Sailfish occupied the Yucatán hotspot throughout the year. January and April are peak months for sailfish fishing off the Yucatán Peninsula, and fish tagged in January and February were available in April either because they remained in the area (Fig. 1d and i) or returned from elsewhere (Fig. 1b and c). Two sailfish tagged in April returned the following January (Fig. 1a and f), while others did not (Fig. 1j and k). Reduced usage of the Campache Bank from July to October (Fig. 3a) significantly correlated with a drop in net primary productivity (Pearson two-sided test = 0.59, p = 0.05), but not with changes in sea surface temperature (SST) (p = 0.4) or chlorophyll-a (p = 0.5).

Between April and June, hotspots also appeared off Veracruz, Mexico, and off Northwest Florida (Fig. 2). Further into the presumed peak spawning months of July and August, sailfish utilized a broader area on the East Mexico Shelf, West Florida Shelf and Straits of Florida, the last of which is a known spawning ground. Utilization of continental shelf hotspots was highest three to four months after peak primary production in the spring (Fig. 3b and c). From October to December, sailfish were located in the Mexico Basin (central GOM), and in the West Atlantic (Fig. 2). Despite use of Mexico Basin peaking in October, elevated use of this hotspot also occurred three months after the spring bloom (Fig. 3d).

### Vertical activity

**Figure 4:**
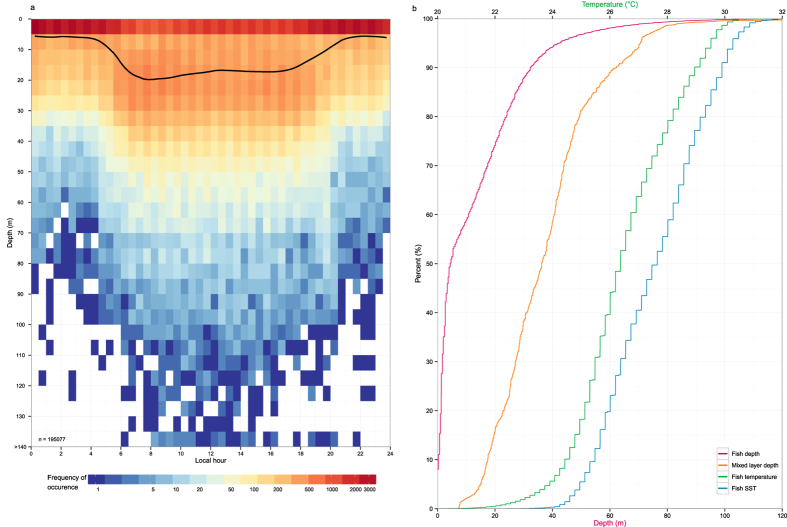
Vertical habitat of tagged sailfish. Depth utilization over 24 hours (**a**), with average depth denoted by a black line. Cumulative percentages (**b**) of sailfish depth (red), temperature (green) and sea surface temperature (blue), and along-track, mixed layer depth (orange). Data were pooled from all tagged fish.

Tagged sailfish occupied predominantly the uppermost portion of the water column (median = 4.4 m, IQR = 19 m). Depth distribution rarely exceeded 100 m (Fig. 4), although maximum depth recorded was 452 m (Table 1). Significant diel differences in depth distribution were exhibited by all sailfish (Fig. 4a), except for two that remained in the Isla Mujeres area during January and April (two-sample Kolmogorov–Smirnov test; Tag 2013–20555, p = 0.08; Tag 2013–20561, p > 0.17). Mean depth during the daytime was 18+18 m and 8+12 m during the nighttime. Overall, sailfish stayed above 10 and 50 m 59% and 97% of the time, respectively (Fig. 4b). Only 5% of temperatures were <24°C, with minimum and median temperatures of 12.3 and 26.4 °C, respectively. Tag-derived SSTs were 22.4–33.9 °C, while 72% fell within 25–29 °C (Fig. 4b).

**Figure 5:**
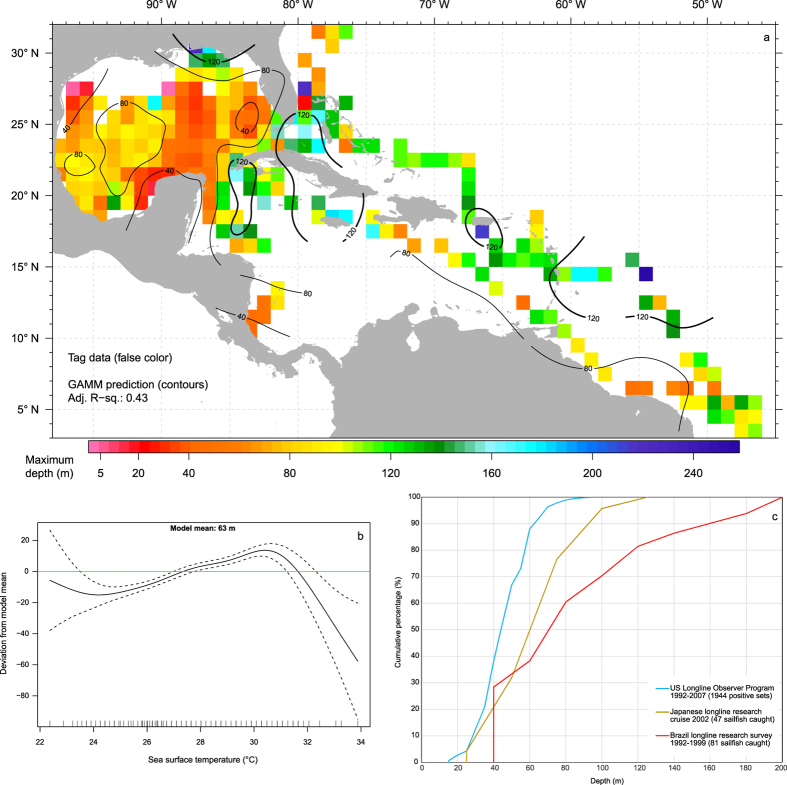
Maximum depth of sailfish. Predictions from the best-fitting generalized additive mixed model (**a**) are represented by contours, while tag observations are binned to generate average values in a 1°x1° grid and plotted in false color; estimated individual effect of sea surface temperature on maximum depth (**b**). Dashed lines show 95% confidence limits. Ticks on x-axis denote values for which there are data. To aid visualization, a horizontal line is added at 0 on the y-axis. Positive values on y-axis mean deeper depth. Depth of longline caught sailfish (**c**) from the US Observer program 1992–2007 (1944 sets)^46^, the Japanese research cruise in 2002 in the tropical Atlantic (47 sailfish)^48^, and small-scale research surveys between 1992–1999 in the southwestern equatorial Atlantic (81 sailfish)^47^. Hook depths were either calculated^46^,^47^ or recorded by time-depth-temperature recorder attached to the hook line^48^. Maps were generated in R^79^ (v2.15.2).

Results from generalized additive mixed modeling identified location and SST as the primary predictors of maximum depth (adjusted R^2^ = 0.43). Month, mixed layer depth, sea surface height deviation and net primary productivity were not found to be significant. Maximum depth of sailfish was between 40 and 80 m in the Gulf of Mexico, and 120 m outside of GOM in the West Atlantic (Fig. 5). Deeper maximum depths occurred off Louisiana, along the Antilles chain, and in areas with SST between 27–31 °C (Fig. 5b). This latitudinal deepening of maximum depth from north to south mirrors depths at which sailfish were caught on longline by the US^46^ and Brazilian^47^,^48^ fleets that operated within their respective hemispheres (Fig. 5c).

**Figure 6:**
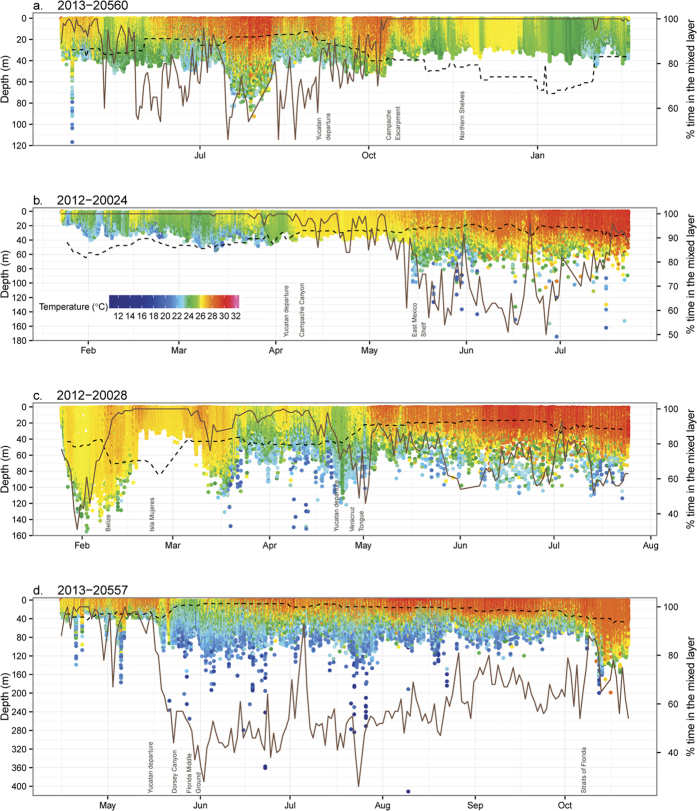
Depth and temperature time series of sailfish. Recovered tags provided data at 2-minute resolution. Mixed layer depth was extracted daily at each track position (dashed line). Percent of daily time spent in the mixed layer is then calculated and plotted on the secondary y-axis (brown line). Labels on the x-axis indicate the general location of sailfish at that time. Notice y-axes are scaled for better visualization.

**Figure 7:**
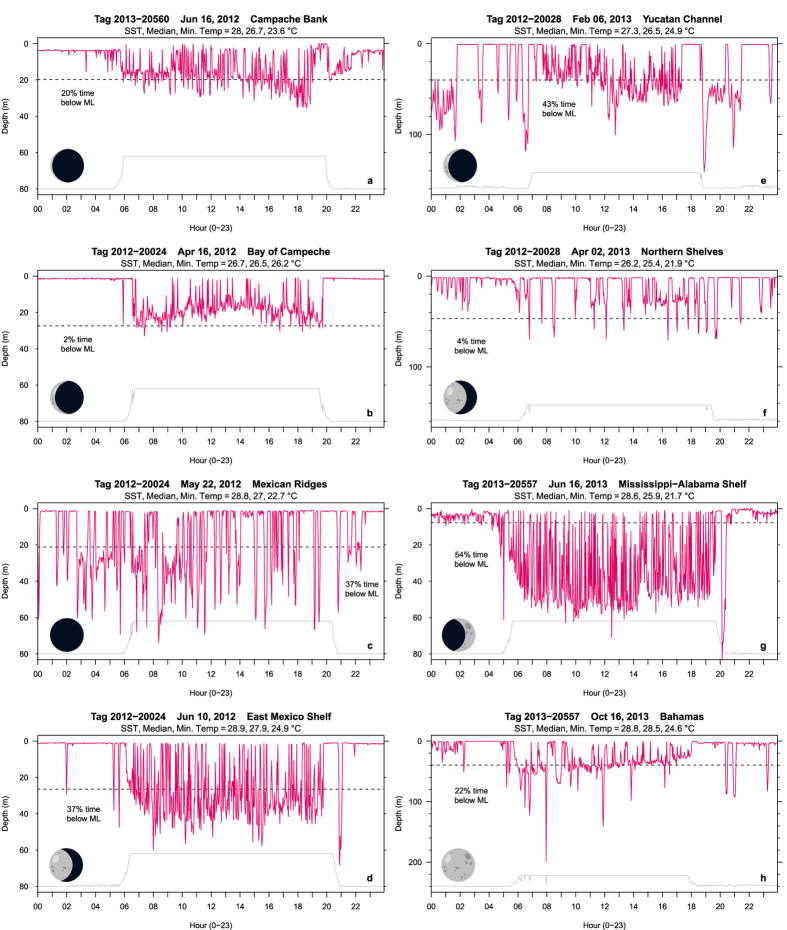
Representative depth profiles of sailfish over 24-hour. Relative light intensity tracks periods of darkness and light, as well as sharp changes in ambient light during sunrise and sunset (gray line). Mixed layer depth is also shown (dotted line). Moon phase is represented by icon. Labels denote additional information on temperature and percent time spent below the mixed layer. Notice y-axes are scaled for better visualization.

Sailfish spent 26–100% (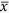 = 81 ± 19%) of their day in the mixed layer, which varied between 7–83 m across locations (Fig. 6). Around the Yucatán Peninsula, sailfish stayed predominantly in the mixed layer during winter and early spring (Fig. 6a and b). As sailfish moved away from the Yucatán, time spent in the mixed layer decreased and varied between 40–80% (Fig. 6b–d). Diel differences in swimming depth were evident, including punctuated descents and ascents during sunrise and sunset (Fig. 7). During the day, sailfish hovered around the base of the mixed layer (Fig. 7a,b,e and h), near the surface (Fig. 7c and f), or deeper than the mixed layer (Fig. 7d and g). Repeated surface excursions were common, especially on the continental shelf during June (e.g., Fig. 7d and g) and July. At night, sailfish remained near the surface, occasionally descending deeper (Fig. 7c) or staying below the mixed layer for greater than an hour (Fig. 7e). Lunar illumination did not correlate with changes in swimming behavior.

## Discussion

This study provides yearlong, fisheries independent information on behavior, horizontal and vertical habitat use of sailfish released from an aggregation site in the Northwest Atlantic. We achieved a high proportion (41%, n = 9) of PSATs remaining attached and reporting after the full year mission. This result surpassed the highest yearlong tag retention rate (<35% from great white sharks, *Carcharodon carcharias*) in a meta-analysis of tag performance^49^. Tagged sailfish moved primarily north to south, and frequented productive coastal areas. They mainly occupied the surface layer above 30 m, and displayed diel depth patterns, with deeper daytime depths, but rarely deeper than 100 m.

### Horizontal habitat

Isla Mujeres, Mexico is clearly a prominent mixing and foraging ground for Atlantic sailfish. Over one year, sailfish dispersed from the Yucatán to multiple, distant regions, presumably to spawn and forage, before returning to the Yucatán coast the following year, some with striking proximity to their release location. Such navigation ability is similar to that of bluefin tuna (*Thunnus thynnus*)^50^ and swordfish (*Xiphias gladius*)^51^. Onset of migration did not appear to be related to SST (Fig. 6), contrary to an earlier hypothesis that migratory movements were driven by water temperature^52^. This study released tagged fish in the Yucatán area at different times of year, over multiple years. Sailfish dispersal was primarily westward in the release year of 2012, northwest and northeastward in 2013, and omnidirectional in 2014. If tags had not remained attached on fish through late winter, we would not have identified fish that traveled to the South American coast before returning to the Gulf of Mexico. Given the diversity in sailfish movement patterns observed across sampling years, it is inadequate to rely upon a small number of tracks to monitor potential shifts in billfish habitat following a major environmental disaster, such as the Deep Horizon oil spill^53^. Since billfishes likely spawn at multiple sites throughout the Atlantic^31^,^54^,^55^, the loss of a particular spawning site may have limited impacts on the populations. Having appropriate baseline information on pelagic animal movements is important for robust vulnerability and impact evaluations^56^.

Eddy frontal zones are a favorable environment for sailfish spawning and larval development^36^,^38^. While PSAT tags alone do not have the capabilities to “observe” and confirm the biological act of spawning, based on the spatiotemporal presence of tagged sailfish (Fig. 2), the East Mexico Shelf is a potential spawning habitat in western GOM, as suggested by the historic presence of “ripe” females^57^. Persistent eddy features (lasting >28 days) are common in this area, especially off Veracruz, Mexico^58^, and sailfish larval abundance increased with Sargassum biomass at eddy features in northern GOM^37^,^38^.

### Combined analysis from satellite and conventional tagging

**Figure 8:**
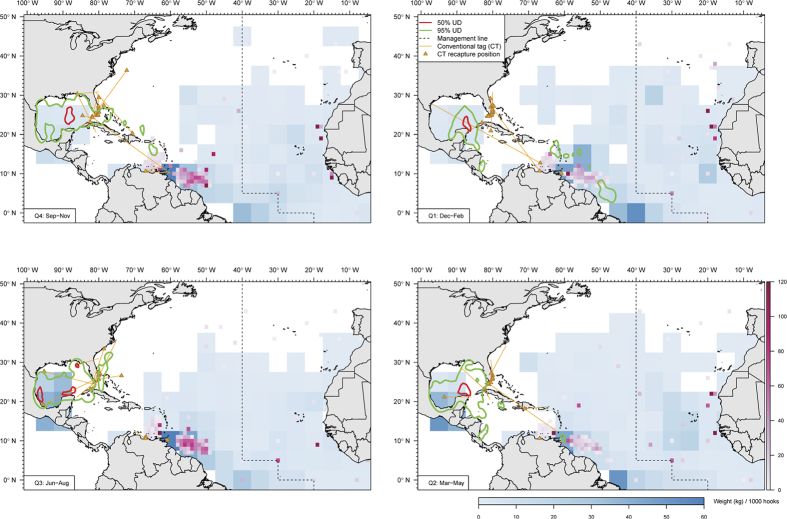
Sailfish longline catch per unit effort (CPUE) distribution. Distribution of longline CPUE (kilograms of sailfish caught per 1,000 hooks) by quarters (Q1: Dec-Feb, Q2: Mar-May, Q3: Jun-Aug, Q4: Sep-Nov) from Task II data reported at 1°x1° (magenta scale) and 5°x5° (blue scale) between 2000 and 2013. Over the same period, recapture positions (orange triangles) for conventional tag deployments (orange lines) were grouped by the quarter in which a tag was recaptured. Only deployments from three to twelve months are included. Utilization distribution (UD) contours (95%, green; 50% red) of tagged sailfish are also shown. Maps were generated n R 79 (v2.15.2).

PSAT-derived movement patterns can be used to interpret conventional tag data^59^, which sailfish have the second-most tag releases and recaptures after Atlantic bluefin tuna^21^,^22^. Seasonal utilization distribution derived from PSATs (Figs 2 and 8) is consistent with conventional tag recaptures of fish at liberty between two and eighteen months (Fig. 8 and Figure S3). Both conventional and PSAT data confirmed that sailfish visited multiple hotspots in the Gulf of Mexico and off Florida within six months’ time. Some sailfish were furthest away from Isla Mujeres or South Florida during January to March, when located, for example, off the South America coast (Fig. 1j), or in the central North Atlantic (Fig. S3).

By analyzing only conventional tag returns, Orbesen, *et al*.^21^ concluded that sailfish are closely associated with coastal regions and have limited dispersals in comparison with blue and white marlin. Among their recaptures, 68% of sailfish were off the Florida East Coast (FEC), where abundance peaked during late fall and early spring, coinciding with a high tag-and-release effort. In comparison, our results confirmed connectivity between the Yucatán Peninsula and FEC (Fig. 1h–j). Moreover, sailfish visited other coastal areas before (e.g., northern GOM) and after (e.g., South America) FEC, all within the same year. Sailfish cyclic, annual migrations from Isla Mujeres integrate different productive areas of the Northwest Atlantic. Individuals can travel as far north as the Sambro Bank, off Nova Scotia (Fig. S3; conventional tag HHM052728, released off Isla Mujeres, 520 days at liberty) as part of their annual migration. At the southern extent, our study observed sailfish movements linking Isla Mujeres and the northeastern coast of Brazil, a journey that took at least three months. Connection to the southeastern coast of Brazil remains undocumented, as PSATs from an earlier study all detached within 51 days post-release^41^.

Consistent with earlier studies, PSAT-tagged sailfish did not exhibit trans-Atlantic movement. Nonetheless, cyclic migrations demonstrated that sailfish of various sizes are capable of traveling long distances that can result in the Atlantic-wide catch distribution of the longline fishery (Fig. 8). Pockets of high CPUE found offshore along 35°W would require sailfish sourced elsewhere, most likely from coastal hotspots. Mixing of the two managed stocks probably occurs in the South Central Atlantic^60^, but elucidating the mixing mechanisms and rates will require a sufficient bounty of year-long tracks to be obtained offshore, not just from coastal areas such as off Isla Mujeres or Florida.

### Vertical habitat

The epipelagic layer, particularly 0–20 m, was heavily utilized by Atlantic sailfish tagged in this and other studies^19^,^20^,^61^. Prince, *et al*.^23^ compared the proportion of time sailfish spent at 0–50 m between the eastern and western Atlantic (97% vs 81%), and attributed the difference to a shallow oxycline in the East that compresses the vertical habitat of pelagic fishes. In this study, tagged sailfish remained in the well-oxygenated West Atlantic, but occupied 0–50 m for 97% of time (Fig. 4), similar to tagged eastern fish^23^. Sailfish also exhibited a broad range of vertical behavior in different areas of the West Atlantic (Figs 5, 6 and 7). Such geographical differences likely reflect varying prey type and availability, biophysical conditions, and life history. Our latest data do not invalidate dissolved oxygen as a key physiological constraint^62^, but rather they illustrate the difficulty in explaining regional differences in vertical habitat use with an environmental variable. To rigorously evaluate habitat associations and assess physiological limits, the sample size for long-term electronic tags must be large enough to provide adequate geographic coverage, and opportunities to observe the diversity of animal behaviors.

Descents to depths around or below the mixed layer (Fig. 7) are likely related to foraging on spawning herring (*Sardinella aurita)* and cigar minnows (*Decapterus punctatus*), which we repeatedly observed during tagging operations. Tagged sailfish undertook more excursions during the day, consistent with Hoolihan, *et al*.^61^, and also with most sailfish (13 out of 14) being caught during daylight on longline targeting swordfish in the Gulf of Mexico^63^. Twilight may represent an opportune time for foraging^64^, as sailfish often descended just before sunrise/ sunset and resurfaced shortly afterward (e.g., Fig. 7e), a behavior also displayed by giant bluefin tuna and other predators in coastal areas^65^.

The sensory and thermal biology of sailfish are important in consideration of fisheries impacts. Sailfish are highly visual predators^66^, but appear capable of foraging in low light. Sailfish were active in nighttime (e.g., Fig. 7c) through all moon phases, similar to fish tracked off Florida^19^, However, under low light, sailfish appear more catchable by drift gillnets, as the artisanal fishery off La Guaira, Venezuela only operates during the new moon^67^.

Sailfish can briefly withstand low temperature (min. 11.1 °C), and deep depth (max. 560 m), where it is dark^20^. This diversity in vertical behavior is reflected in the broad spectrum of prey items identified in stomach content analyses, which include fish, cephalopod and crustaceans^28^,^47^,^68^. Off northeastern Brazil, sailfish prey heavily on Atlantic pomfret (*Brama brama*), flying gurnard (*Dactylopterus volitans*) and snake mackerel (*Gempylus serpens*), along with 17 other identified fish taxa^47^. Although not evident from the depth distribution of Atlantic sailfish (Fig. 4), black swallowers (*Kali parri*), a mesopelagic species^69^, was also found in their diet^47^. Like Atlantic tunas^70^, sailfish prey on cephalopods while offshore, and are not confined to productive coastal areas, as shown by longline catch distribution (Fig. 8). PSAT tagging and sampling of sailfish in pelagic regions would provide additional insights into their trophic ecology.

Our electronic tagging results have provided a baseline and more complete understanding of habitat utilization of sailfish in the western Atlantic. Combined electronic and conventional tagging data demonstrated seasonal sailfish migration connecting multiple coastal areas with open ocean transits. Pelagic transits in other parts of the Atlantic could be important in connecting sailfish hotspots across the ocean basin. Understanding these movements will help address uncertainties in mixing and stock structure in assessment, and ultimately, broader spatiotemporal information may improve management of Atlantic sailfish.

## Materials and Methods

### Tagging

Thirty-four PSATs (30 X-Tag; Microwave Telemetry, Inc. and 4 MiniPAT; Wildlife Computers Inc.) were deployed on adult sailfish (mean ± SD = 162 ± 11 cm LJFL) between 2012 and 2014 by rod and reel aboard F/V Chachalaca and F/V Lily off Isla Mujeres, Mexico. Upon capture, an individual that was undamaged, assessed to be in good condition, and large enough for tagging (i.e., >35 kg), was brought on board and placed on a wet, padded vinyl mat. A wet soft cloth was immediately placed over its eyes to reduce stress and a seawater hose was inserted in its mouth to irrigate the gills. Tag tethers and anchors were constructed according to materials and methods we developed for bluefin tuna tagging^71^,^72^, with darts implanted in the musculature at the anterior dorsal region. LJFL was measured to the nearest centimeter and a fin clip was taken and archived for genetic analysis. Fish were immediately released and, in many cases, underwater footage of tagged fish was obtained to document post release behavior. Tag and release was carried out in accordance with relevant guidelines and regulations. The experimental protocol (#2013–0041) was approved by University of Massachusetts Institutional Animal Care and Use Committee.

PSATs were programmed to record relative light level, external temperature and pressure (depth) for 4 to 6 months (per funders’ request) in the first year of study, and 12 months in subsequent years. Physically recovered X-Tags contained the full resolution time series at 2-minute resolution; otherwise data were sub-sampled by manufacturer routines for transmission through the Argos satellites. Transmitted formats included estimated sunrise and sunset times, daily minimum and maximum depths and temperatures, and depth and temperature records available at the 15-minute marks (:00,15,30,45) of the hour. MiniPATs sampled every 60 seconds, and were configured to transmit time series data at 5 and 7.5-minute resolution, light levels at times of sunset and sunset, daily summaries of depth and temperature, and time-at-temperature and depth histograms in 6-hour blocks. Temperature bins were 8, 12, 14, 16, 18, 20, 22, 26, 26, 28, 30 & >30 °C, and depth bins were 0, 2, 10, 20, 50, 75, 100, 125, 150, 200, 300 & >300 m. All tags had a constant depth failsafe release set at 3 (MiniPAT) or 4 (X-Tag) days, which would indicate post-release mortality or tag shedding. Returned data were imported into, and managed through Tagbase^73^.

### Geolocation

For X-Tags, positions generated by manufacturer software were refined using a state-space Kalman filter model with SST matching, *Ukfsst*^74^ with NOAA Optimum Interpolation SST V2 (www.esrl.noaa.gov/psd/data/gridded/data.noaa.oisst.v2.html). For MiniPATs, a state-space Kalman filter model, *TrackIt* was used to estimate positions based on transmitted light data and SST^75^,^76^. To further refine positions that fell on land, bathymetric correction was applied^50^. Since there were gaps in transmitted data, refined tracks were ‘regularized’ to a daily resolution using the R package ‘*crawl*’^77^. This final step used positional error estimates from the state-space models as a priori variance. From the final tracks and their associated error, utilization distributions (UDs) were generated monthly and quarterly^78^. We defined the area inside a 50% UD contour as a utilization hotspot, and simply referred to as “hotspot”.

For sailfish that were >30 days at liberty, we defined residency in the Yucatán Peninsula area as the duration of time before an individual moved off the continental shelf, or west of 90°W.

### Tag-recorded water column data

We defined depth and temperature records as day or night, based on local sunset and sunrise times calculated from estimated positions using the ‘sunriset’ function in the R^79^ (v2.15.2) package ‘maptools’ (v0.8–23; cran.r-project.org/web/packages/maptools). Data were summarized daily, and differences between SST and ambient temperatures (mean, minimum) recorded by the fishes’ tags were also calculated. Means (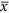) are reported plus or minus standard deviation (SD) unless otherwise indicated.

### Environmental parameters

To model sailfish habitat, we obtained VIIRS SST (triple-window algorithm) and chlorophyll-a (OCI algorithm) data from NASA OceanColor Web (oceancolor.gsfc.nasa.gov), mixed layer depth from NCEP Global Ocean Data Assimilation System through NOAA ERDDAP (upwell.pfeg.noaa.gov/erddap/griddap/noaa_pmel_2e50_792a_c159.html), net primary productivity (Vertically Generalized Production model) data from Oregon State University Ocean Productivity (www.science.oregonstate.edu/ocean.productivity), and sea surface height (SSH) data from the HYbrid Coordinate Ocean Model (HYCOM) of Center for Ocean-Atmospheric Prediction Studies (hycom.org/dataserver/glb-reanalysis). Bathymetric values were obtained from Smith & Sandwell Topography (0.0167° resolution, version 11.1). Along each track point, environmental data were extracted for the neighborhood bounded by the 95% confidence region around a position estimate to generate a mean value. This was performed to account for differing spatial resolution of environmental datasets.

To estimate the mean position of the Loop Current and its associated eddy features, SSH data were averaged monthly, quarterly and annually from 2012 to 2014. Loop Current features were tracked using the 0.17-m contour^80^.

Lunar illumination (aa.usno.navy.mil/data/docs/MoonFraction.php), uncorrected for cloud cover, was tested for correlation with daily mean and maximum depth during nighttime.

### Generalized additive mixed model (GAMM)

We used GAMMs to predict maximum depth of sailfish. Since observations were repeated measures collected from the same individuals, we modeled individual fish as a random effect. We incorporated the following explanatory variables as fixed effects: location, month, SST, mixed layer depth, sea surface height deviation and net primary productivity. We accounted for spatial patterns by explicitly modeling location as a fixed effect.

Models were constructed with the R package, *mgcv* (v1.7–22) using the Gaussian family with an identity-link function^81^. Other than month, explanatory variables were modeled as continuous variables and smoothed. Smoothing functions were chosen automatically and evaluated manually using the ‘gam.check’ function. We adopted the “top-down” model selection strategy^82^ by starting with a “beyond optimal” model, and subsequently dropping explanatory variables to arrive at the final model. The starting model is formulated as follows:


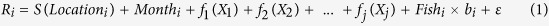


Where *R*_i_ is the matrix of vertical activity for *Fish*_*i*_, and *i* = 1, …, number of individual fish *n*;

*S* is a smoothing function for location;

*Location*_*i*_ is the matrix of longitude and latitude for *Fish*_*i*_;

*Month*_*i*_ is the matrix of month of observation for *Fish*_*i*_;

*f*_1_ … *f*_*j*_ are smoothing functions for environmental variable *j*, and j = 1, …, number of different environmental variables;

*X*_1_ … *X*_*j*_ are matrices of environmental variable *j* for *Fish*_*i*_;

*b*_*i*_~ *N*(0, D) and ε ~ *N*(0, *σ*^2^) where D and *σ*^2^ are variances, and *b*_1_, … *b*_*n*_, ε are independent.

We selected the final model by minimizing both the Akaike Information Criterion (AIC) and Bayesian Information Criterion (BIC) scores. Candidate predictors that were statistically significant at the 0.05 level were retained during the selection process. Finally, we evaluated the models by checking diagnostic plots on fitted data and residuals (Fig. S4).

### Fisheries data

To evaluate movements from fish tagged in coastal areas in relation to the pelagic realm, as indicated by longline fishery catches in the North Atlantic, we obtained conventional tag and fisheries data (Task II catch/effort; version Nov 2015) from ICCAT (www.iccat.int/en/accesingdb.htm). We extracted longline (LL) data available at two spatial resolutions, 5° × 5° and 1° × 1° for the years 2000–2013 to calculate an average catch-per-unit effort (CPUE; weight of fish in kilograms per 1000 hooks) at each grid square. Changes in distribution of quarterly CPUE were checked against 95% and 50% UD contours.

The ICCAT conventional tag database contains sailfish release and recapture records only to 2011, so we incorporated records from the Billfish Foundation tag and release database (www.tagbillfish.org) for the later years. In addition, release and reporting positions from an earlier PSAT study^36^ were included as conventional tags. To map seasonal movements, we only included trajectories from tags recovered after 2 to 18 months at liberty.

## Additional Information

**How to cite this article**: Lam, C. H. *et al*. Sailfish migrations connect productive coastal areas in the West Atlantic Ocean. *Sci. Rep.* **6**, 38163; doi: 10.1038/srep38163 (2016).

**Publisher's note:** Springer Nature remains neutral with regard to jurisdictional claims in published maps and institutional affiliations.

## Supplementary Material

Supplementary Information

## Figures and Tables

**Table 1:** Tagging and data summary for adult Atlantic sailfish, *Istiophorus platypterus*.

Tag ID	Model	Mission (months)	Tag Date	Report Date	At liberty (days)	Depth & temperature data transmitted (%)	Tag Latitude (°N)	Tag Longitude (°W)	Report Latitude (°N)	Report Longitude (°W)	Distance between tag & report locations (km)	Furthest displacement straight line distance (km)	Lower jaw fork length (cm)	Max. depth (m)	Min. temperature (°C)	Max. temperature (°C)	Last Date in the Yucatan area	Residence (days)
2012–11P0221^c^	Mini-PAT	4	24-Jan-2012	20-May-2012	117	0	21.28	86.67					170					
2012–11P0222	Mini-PAT	4	25-Jan-2012	10-Feb-2012	16	73	21.28	86.67	21.89	87.15	84	84	163	40	19.8	26.4		
2012–11P0223	Mini-PAT	4	25-Jan-2012	6-Feb-2012	12	79	21.28	86.67	22.61	86.97	150	150	160	168	20.0	26.6		
2012–11P0225	Mini-PAT	4	25-Jan-2012	31-May-2012	127	40	21.28	86.67	22.76	90.80	457	457	155	64	19.6	28.8	31-May-2012	128
2012–20021^a^,^d^	X-Tag	6	23-Jan-2012	13-Feb-2012	1	100	21.28	86.67	22.78	86.79	167		150	26	25.3	26.4		
2012–20022	X-Tag	6	23-Jan-2012				21.28	86.67					163					
2012–20023	X-Tag	6	23-Jan-2012				21.48	86.67					175					
2012–20024^a^	X-Tag	6	23-Jan-2012	23-Jul-2012	182	63	21.67	86.67	25.44	96.53	1089	1102	157	175	18.3	31.3	5-Apr-2012	73
2012–20025^d^	X-Tag	6	23-Jan-2012	7-Mar-2012	34	94	21.67	86.67	22.19	87.99	148	251	160	81	19.7	29.1	7-Mar-2012	44
2012–20026	X-Tag	6	25-Jan-2012	25-Jul-2012	182	60	21.67	86.67	22.18	89.37	285	698	168	161	19.4	30.5	13-Mar-2012	48
2012–20027	X-Tag	6	24-Jan-2012	15-Jun-2012	143	2	21.48	86.67	22.60	89.98	363	904	173	231	20.2	29.1	20-Apr-2012	87
2012–20028^a^	X-Tag	6	25-Jan-2012	25-Jul-2012	182	66	21.48	86.67	21.70	92.82	637	1062	160	155	17.8	30.7	23-Apr-2012	89
2013–20555^a^	X-Tag	12	14-Apr-2013	28-Apr-2013	14	100	21.38	86.60	25.26	84.93	462	462	171	71	21.2	31.7		
2013–20556	X-Tag	12	14-Apr-2013				21.43	86.67					165					
2013–20557^a^	X-Tag	12	15-Apr-2013	30-Oct-2013	198		21.45	86.65	26.48	68.48	1929	1929	172	411	12.3	31.1	16-May-2012	
2013–20558	X-Tag	12	16-Apr-2013	16-Apr-2014	365	49	21.45	86.61	24.17	86.23	304	4768	147	452	13.8	31.1	20-Apr-2013	4
2013–20559	X-Tag	12	16-Apr-2013				21.48	86.63					168					
2013–20560^b^	X-Tag	12	16-Apr-2013	19-Feb-2014	309		21.51	86.65	21.76	86.67	28	394	183	117	19.9	30.9	10-Sep-2013	147
2013–20561	X-Tag	12	17-Apr-2013	24-Jan-2014	282	61	21.40	86.65	22.21	88.36	198	870	164	183	18.4	31.9	7-Jul-2013	81
2013–20562^c^	X-Tag	12	17-Apr-2013	17-Apr-2014	365	0	21.40	86.65	20.89	86.82	59		171					
2013–20563	X-Tag	12	17-Apr-2013	12-May-2013	25	99	21.40	86.65	25.25	86.34	428	428	176	312	18.1	30.3		
2013–20564	X-Tag	12	17-Apr-2013	20-Jun-2013	64	98	21.40	86.65	27.94	89.32	773	773	169	188	16.8	33.1	9-Jun-2013	53
2013–20565^a^,^e^	X-Tag	12	18-Apr-2013	18-Apr-2014	365		21.43	86.68	22.77	88.69	255	570	161	261	14.8	33.3	24-Apr-2013	6
2013–20566	X-Tag	12	18-Apr-2013				21.43	86.68					165					
2014–20129	X-Tag	12	21-Feb-2014	9-Jan-2015	322	21	21.07	86.07	21.79	88.27	242	1112		312	15.9	31.3	21-Apr-2014	59
2014–20259	X-Tag	12	12-Feb-2014	11-Feb-2015	364	<1	21.98	86.98	22.45	87.82	101	435	137	43				
2014–20260	X-Tag	12	10-Feb-2014	12-Oct-2014	244	56	21.98	86.98	27.71	96.79	1177	1177	170	151	18.3	30.9	18-Apr-2014	67
2014–20261	X-Tag	12	12-Feb-2014	1-Apr-2014	48	93	21.97	87.37	20.40	91.84	496	518	147	48	22.5	28.0	26-Feb-2014	14
2014–20262	X-Tag	12	12-Feb-2014	11-Feb-2015	364	29	21.97	87.37	21.91	87.64	29	1548	170	301	13.5	31.5	14-Mar-2014	30
2014–20263	X-Tag	12	10-Feb-2014				21.98	86.98					173					
2014–20264	X-Tag	12	20-Feb-2014	16-Jul-2014	146	12	21.83	86.83	25.58	87.85	428	428	150	436	15.2	30.3	23-Apr-2014	62
2014–20265^d^	X-Tag	12	10-Feb-2014	20-Mar-2014	35	98	21.97	86.98	22.20	87.32	43	167	142	65	22.0	27.7	20-Mar-2014	38
2014–20266^a^	X-Tag	12	28-Jan-2014	6-Feb-2014	9	100	21.92	86.92	22.34	88.28	148	148	152	27	23.2	32.1		
2014–20267	X-Tag	12	28-Jan-2014	28-Jan-2015	365	41	21.92	86.92	22.05	86.40	56	1273	152	285	16.4	31.1	1-Feb-2014	4

^a^Tag physically recovered.

^b^Recaptured before mission end date.

^c^Transmitted data insufficient for decoding.

^d^Predated.

^e^Tag did not report, popoff position estimated.

## References

[b1] Alió J. J. Recreational fishery component of the Caribbean large marine ecosystem, large pelagic fisheries case study: southern Caribbean area (Venezuela with notes from Colombia). CRFM Research Paper Collection 7, 1–26 (2012).

[b2] Brinson A. A., Alcala A., Die D. J. & Shivlani M. Contrasting socioeconomic indicators for two fisheries that target Atlantic billfish: Southeast Florida recreational charter boats and Venezuelan artisanal gill-netters. Bull. Mar. Sci. 79, 635–645 (2006).

[b3] Cox A. Socioeconomics of billfish sportfishing in Isla Mujeres, Mexico– an evaluation of the relationship between willingness to pay and catch per unit effort in *Proceedings of the Gulf and Caribbean Fisheries Institute*. 1–11 (2010).

[b4] Martinez M. A. & Gonzalez M. E. A review of the recreational fishery for the Atlantic sailfish *Istiophorus albicans* in Cozumel island, Quintana Roo, Mexico in *Report of the Second ICCAT Billfish workshop. Inter. Comm. Cons, Atl. Tunas C*. 278–286 (1994).

[b5] Agnissan J.-P. A., Kone A., Konan S. K. & N'Da K. Dynamics of the exploited population of *Istiophorus albicans* (Latreille, 1804, Istiophoridae) by marine artisanal fishing in Côte d'Ivoire (West Africa). Int. J. Innovation Appl. Stud. 7, 382–392 (2014).

[b6] Arocha F. . Billfish catch in the Venezuelan artisanal off-shore pelagic longline fishery: past and present (1986–2013). Col. Vol. Sci. Pap. ICCAT 71, 2203–2216 (2015).

[b7] Brinson A. A., Die D. J., Bannerman P. O. & Diatta Y. Socioeconomic performance of West African fleets that target Atlantic billfish. Fish. Res. 99, 55–62, doi: 10.1016/j.fishres.2009.04.010 (2009).

[b8] Diatta Y., Die D. J. & Fitchett M. Relative abundance indices for sailfish from the artisanal fleet from Senegal. Col. Vol. Sci. Pap. ICCAT 65, 1725–1739 (2010).

[b9] Anon. Report of the 2009 sailfish stock assessment. Col. Vol. Sci. Pap. ICCAT 65, 1507–1632 (2010).

[b10] Restrepo V., Prince E. D., Scott G. P. & Uozumi Y. ICCAT stock assessments of Atlantic billfish. Mar. Freshwater Res. 54, 361–367, doi: 10.1071/mf02057 (2003).

[b11] National Oceanic and Atmospheric Administration. Fisheries of the United States; Billfish Conservation Act of 2012 implementing regulations; proposed rule. in *78 FR 20291* 20291–20292 (2013).

[b12] National Oceanic and Atmospheric Administration. Atlantic highly migratory species (HMS); pelagic longline fishery; final rule. in *69 FR 40734* 40734–40758 (2004).

[b13] National Oceanic and Atmospheric Administration. Atlantic highly migratory species; bluefin tuna bycatch reduction in the Gulf of Mexico pelagic longline fishery; final rule. in *76 FR 18653* 18653–18661 (2011).

[b14] MAC. Resolución MAC/DGS/104. Gaceta Oficial No. 34.449. Caracas, 17 Abril 1990 (1990).

[b15] MPC. Resolución MPC/DM/No. 020, Gaceta Oficial No. 5.438 (extraordinaria). Caracas, 08 Febrero 2000 (2000).

[b16] Kerstetter D. W. & Graves J. E. Postrelease survival of sailfish caught by commercial pelagic longline Gear in the southern Gulf of Mexico. N. Am. J. Fish. Manage. 28, 1578–1586, doi: 10.1577/m07–202.1 (2008).

[b17] Walter J. F., Orbesen E. S., Liese C. & Serafy J. E. Can circle hooks improve western Atlantic sailfish, *Istiophorus platypterus*, populations?". Bull. Mar. Sci. 88, 755–770, doi: 10.5343/bms.2011.1072 (2012).

[b18] Lima C. W. *Evaluating the impact of stock structure uncertainty in stock assessments of sailfish in the Atlantic Ocean* Doctoral thesis, The College of William and Mary (2012).

[b19] Kerstetter D. W., Bayse S. M., Fenton J. L. & Graves J. E. Sailfish habitat utilization and vertical movements in the southern Gulf of Mexico and Florida Straits. Mar. Coast. Fish. 3, 353–365 (2011).

[b20] Mourato B. L. . Short-term movements and habitat preferences of sailfish, *Istiophorus platypterus* (Istiophoridae), along the southeast coast of Brazil. Neotrop. Ichthyol. 12, 861–870, doi: 10.1590/1982–0224–20130102 (2014).

[b21] Orbesen E. S. . Transboundary movement of Atlantic istiophorid billfishes among international and US domestic management areas inferred from mark-recapture studies. Mar. Fish. Rev. 70, 14–23 (2008).

[b22] Orbesen E. S., Snodgrass D., Hoolihan J. P. & Prince E. D. Updated US conventional tagging database for Atlantic sailfish (1955–2008), with comments on potential stock structure. Col. Vol. Sci. Pap. ICCAT 65, 1692–1700 (2010).

[b23] Prince E. D. . Ocean scale hypoxia-based habitat compression of Atlantic istiophorid billfishes. Fish. Oceanogr. 19, 448–462, doi: 10.1111/j.1365–2419.2010.00556.x (2010).

[b24] Bangma J. *Contemporary population structure and historical demography of sailfish (Istiophorus platypterus) in the Atlantic Ocean* Master thesis, Texas A&M University (2006).

[b25] McDowell J. R. & Graves J. E. A genetic perspective on Atlantic sailfish stock structure. Col. Vol. Sci. Pap. ICCAT 54, 805–810 (2002).

[b26] Dingle H. Migration: the biology of life on the move. (Oxford University Press, USA, 2014).

[b27] Arocha F. & Marcano L. A. Life history characteristics of blue marlin, white marlin, and sailfish from the eastern Caribbean Sea and adjacent waters. In Proceedings of the Fourth World Fisheries Congress: Reconciling Fisheries with Conservation. (eds J. Nielsen . ) 587–597 (2008).

[b28] Jolley J. W. The biology and fishery of Atlantic sailfish Istiophonrs platypterus, from Southeast Florida. 35 (1977).

[b29] Mourato B. L. . Preliminary analysis of gonad development, spawning period, sex ratio and length at first sexual maturity of sailfish, *Istiophorus platypterus* in Brazilian coast. Col. Vol. Sci. Pap. ICCAT 64, 1927–1940 (2009).

[b30] deSylva D. P. & Breder P. R. Reproduction, gonad histology, and spawning cycles of North Atlantic billfishes (Istiophoridae). Bull. Mar. Sci. 60, 668–697 (1997).

[b31] Richardson D. E. . Sailfish (*Istiophorus platypterus*) spawning and larval environment in a Florida Current frontal eddy. Prog. Oceanogr. 82, 252–264, doi: 10.1016/j.pocean.2009.07.003 (2009).

[b32] Gehringer J. W. Observations on the development of the Atlantic sailfish *Istiophorus americanus* (Cuvier): with notes on an unidentified species of Istiophorid In Fishery Bulletin of the Fish and Wildlife Service Vol. 57 Fishery bulletin (US Fish and Wildlife Service, 1956).

[b33] Post J. T., Serafy J. E., Ault J. S., Capo T. R. & deSylva D. P. Field and laboratory observations on larval Atlantic sailfish (*Istiophorus platypterus*) and swordfish (*Xiphias gladius*). Bull. Mar. Sci. 60, 1026–1034 (1997).

[b34] Serafy J. E., Cowen R. K., Paris C. B., Capo T. R. & Luthy S. A. Evidence of blue marlin, *Makaira nigricans*, spawning in the vicinity of Exuma Sound, Bahamas. Mar. Freshwater Res. 54, 299–306, doi: 10.1071/mf01273 (2003).

[b35] Voss G. L. A contribution to the life history and biology of the sailfish, *Istiophorus americanus* Cuv. and Val., in Florida waters. Bull. Mar. Sci. 3, 206–240 (1953).

[b36] Richardson D. E., Cowen R. K., Prince E. D. & Sponaugle S. Importance of the Straits of Florida spawning ground to Atlantic sailfish (*Istiophorus platypterus*) and blue marlin (*Makaira nigricans*). Fish. Oceanogr. 18, 402–418, doi: 10.1111/j.1365–2419.2009.00520.x (2009).

[b37] Brown-Peterson N. J., Franks J. S., Comyns B. H. & McDowell J. Do blue marlin spawn in the northern Gulf of Mexico in Proceedings of the Gulf and Caribbean Fisheries Institute. 372–378 (Gulf and Caribbean Fisheries Institute, 2008).

[b38] Rooker J. R. . Distribution and habitat associations of billfish and swordfish larvae across mesoscale features in the Gulf of Mexico. Plos One 7, doi: 10.1371/journal.pone.0034180 (2012). PMC3324529 22509277

[b39] Simms J. R., Rooker J. R., Holt S. A., Holt G. J. & Bangma J. Distribution, growth, and mortality of sailfish (*Istiophorus platypterus*) larvae in the northern Gulf of Mexico. Fish. Bull. 108, 478–490 (2010).

[b40] Leyva-Cruz E., Vásquez-Yeomans L., Carrillo L. & Valdez-Moreno M. Identifying pelagic fish eggs in the Southeast Yucatan Peninsula using DNA barcodes. Genome, doi: 10.1139/gen-2015–0151 (2016). 27753507

[b41] Mourato B. L. . Spatio-temporal trends of sailfish, *Istiophorus platypterus* catch rates in relation to spawning ground and environmental factors in the equatorial and southwestern Atlantic Ocean. Fish. Oceanogr. 23, 32–44, doi: 10.1111/fog.12040 (2014).

[b42] Pimenta E. G., Lima G., Cordeiro C. J., Tardelli M. & Amorim A. Reproduction and stomach content analysis of sailfish, *Istiophorus platypterus*, off Rio de Janeiro State, RJ, Brazil. Col. Vol. Sci. Pap. ICCAT 58, 1589–1596 (2005).

[b43] Martínez-López B. & Zavala-Hidalgo J. Seasonal and interannual variability of cross-shelf transports of chlorophyll in the Gulf of Mexico. J. Marine Syst. 77, 1–20 (2009).

[b44] Merino M. Upwelling on the Yucatan Shelf: hydrographic evidence. J. Marine Syst. 13, 101–121 (1997).

[b45] Zavala-Hidalgo J., Gallegos-García A., Martínez-López B., Morey S. L. & O’Brien J. J. Seasonal upwelling on the Western and Southern Shelves of the Gulf of Mexico. Ocean Dyn. 56, 333–338, doi: 10.1007/s10236–006–0072–3 (2006).

[b46] Goodyear C. P. & Bigelow K. Preliminary explorations of CPUE standardization of the US longline observer sailfish data using STATHBS. Col. Vol. Sci. Pap. ICCAT 65, 1713–1724 (2010).

[b47] Junior T. V., Vooren C. M. & Lessa R. P. Feeding habits of four species of Istiophoridae (Pisces : Perciformes) from northeastern Brazil. Environ. Biol. Fishes 70, 293–304, doi: 10.1023/B:EBFI.0000033345.53182.b9 (2004).

[b48] Yokawa K., Saito H., Kanaiwa M. & Takeuchi Y. Vertical distribution pattern of CPUE of Atlantic billfishes and associated species estimated using longline research data. Bull. Mar. Sci. 79, 623–634 (2006).

[b49] Musyl M. K. . Performance of pop-up satellite archival tags. Mar. Ecol. Prog. Ser. 433, 1–U58, doi: 10.3354/meps09202 (2011).

[b50] Galuardi B. . Complex migration routes of Atlantic bluefin tuna (*Thunnus thynnus*) question current population structure paradigm. Can. J. Fish. Aquat. Sci. 67, 966–976, doi: 10.1139/f10–033 (2010).

[b51] Neilson J. D. . Investigations of horizontal movements of Atlantic swordfish Using pop-up satellite archival tags In Tagging and Tracking of Marine Animals with Electronic Devices (eds Jennifer L. Nielsen .) 145–159 (Springer Netherlands, 2009).

[b52] Hoolihan J. P. Sailfish movement in the Arabian Gulf: a summary of tagging efforts. Mar. Freshwater Res. 54, 509–513, doi: 10.1071/mf01252 (2003).

[b53] Rooker J. R. . Spatial, temporal, and habitat-related variation in abundance of pelagic fishes in the Gulf of Mexico: potential implications of the deepwater horizon oil spill. Plos One 8, e76080, doi: 10.1371/journal.pone.0076080 (2013). 24130759 PMC3794940

[b54] Luckhurst B. E. & Arocha F. Evidence of spawning in the southern Sargasso Sea of fish species managed by ICCAT albacore tuna, swordfish and white marlin. Col. Vol. Sci. Pap. ICCAT, SCRS/111/2015 (2015).

[b55] Schmidt R. F., Rodrigues T., Pimenta E. G., Hilsdorf A. W. S. & Amorim A. F. Prelimilary occurrence of istiophoridae larvae (Perciformes, Xiphioidei) in southern Brazil. Col. Vol. Sci. Pap. ICCAT 71, 2256–2261 (2015).

[b56] Bjorndal K. A. . Better science needed for restoration in the Gulf of Mexico. Science 331, 537–538, doi: 10.1126/science.1199935 (2011). 21292956

[b57] Baughman J. L. Notes on the Sailfish, *Istiophorus americanus* (Lacépède), in the Western Gulf of Mexico. Copeia 1941, 33–37 (1941).

[b58] Faghmous J. H. . A daily global mesoscale ocean eddy dataset from satellite altimetry. Sci. Data 2, 150028, doi: 10.1038/sdata.2015.28 (2015). 26097744 PMC4460914

[b59] Neilson J. D. . Seasonal distributions and migrations of Northwest Atlantic swordfish: inferences from integration of pop-up satellite archival tagging studies. PLoS ONE 9, e112736, doi: 10.1371/journal.pone.0112736 (2014). 25401964 PMC4234629

[b60] Mourato B. L. . First observations of migratory movements and habitat preference of Atlantic sailfish, *Istiophorus platypterus*, in the southwestern Atlantic Ocean. Col. Vol. Sci. Pap. ICCAT 65, 1740–1747 (2010).

[b61] Hoolihan J. P., Luo J., Goodyear C. P., Orbesen E. S. & Prince E. D. Vertical habitat use of sailfish (*Istiophorus platypterus*) in the Atlantic and eastern Pacific, derived from pop-up satellite archival tag data. Fish. Oceanogr. 20, 192–205, doi: 10.1111/j.1365–2419.2011.00577.x (2011).

[b62] Brill R. W. & Lutcavage M. E. Understanding environmental influences on movements and depth distributions of tunas and billfishes can significantly improve population assessments In Island in the Stream: Oceanography and Fisheries of the Charleston Bump Vol. 25 American Fisheries Society Symposium (ed G. R. Sedberry ) 179–198 (2001).

[b63] Kerstetter D. W. & Graves J. E. Effects of circle versus J-style hooks on target and non-target species in a pelagic longline fishery. Fish. Res. 80, 239–250, doi: 10.1016/j.fishres.2006.03.032 (2006).

[b64] Lam C. H., Kiefer D. A. & Domeier M. L. Habitat characterization for striped marlin in the Pacific Ocean. Fish. Res. 166, 80–91 (2015).

[b65] Lutcavage E. M. . Tracking adult North Atlantic bluefin tuna (*Thunnus thynnus*) in the northwestern Atlantic using ultrasonic telemetry. Mar. Bio. 137, 347–358, doi: 10.1007/s002270000302 (2000).

[b66] Fritsches K. A., Partridge J. C., Pettigrew J. D. & Marshall N. J. Colour vision in billfish. Philos. Trans. R. Soc. Lond., B, Biol. Sci. 355, 1253–1256, doi: 10.1098/rstb.2000.0678 (2000). 11079409 PMC1692849

[b67] Arocha F., Ortiz M., Bárrios A., Debrot D. & Marcano L. A. Catch rates for sailfish (*Istiophorus albicans*) from the small scale fishery off La Guaira, Venezuela: Period 1991–2007. Col. Vol. Sci. Pap. ICCAT 64, 1844–1853 (2009).

[b68] Cherel Y., Sabatie R., Potier M., Marsac F. & Menard F. New information from fish diets on the importance of glassy flying squid (*Hyaloteuthis pelagica*) (Teuthoidea: Ommastrephidae) in the epipelagic cephalopod community of the tropical Atlantic Ocean. Fish. Bull. 105, 147–152 (2007).

[b69] Hartel K. E., Kenaley C. P., Galbraith J. K. & Sutton T. T. Additional records of deep-sea fishes from off greater New England. Northeast. Nat. 15, 317–334, doi: 10.1656/1092–6194–15.3.317 (2008).

[b70] Logan J. M. . Contribution of cephalopod prey to the diet of large pelagic fish predators in the central North Atlantic Ocean. Deep Sea Res., Part II 95, 63–73 (2012).

[b71] Wilson S. G. . Movements of bluefin tuna (Thunnus thynnus) in the northwestern Atlantic Ocean recorded by pop-up satellite archival tags. Mar. Biol. 146, 409–423 (2005).

[b72] Lutcavage M. E., Brill R. W., Skomal G. B., Chase B. C. & Howey P. W. Results of pop-up satellite tagging of spawning size class fish in the Gulf of Maine: do North Atlantic bluefin tuna spawn in the mid-Atlantic? Can. J. Fish. Aquat. Sci. 56, 173–177 (1999).

[b73] Lam C. H. & Tsontos V. M. Integrated management and visualization of electronic tag data with Tagbase. Plos One 6, e21810, doi: 10.1371/journal.pone.0021810 (2011). 21750734 PMC3130046

[b74] Lam C. H., Nielsen A. & Sibert J. R. Improving light and temperature based geolocation by unscented Kalman filtering. Fish. Res. 91, 15–25 (2008).

[b75] Nielsen A. & Sibert J. R. State-space model for light-based tracking of marine animals. Can. J. Fish. Aquat. Sci. 64, 1055–1068 (2007).

[b76] Lam C. H., Nielsen A. & Sibert J. R. Incorporating sea-surface temperature to the light-based geolocation model TrackIt. Mar Ecol Prog Ser 419, 71–84 (2010).

[b77] Johnson D. S., London J. M. & Lea M. A. & Durban, J. W. Continuous-time correlated random walk model for animal telemetry data. Ecology 89, 1208–1215, doi: 10.1890/07–1032.1 (2008). 18543615

[b78] Galuardi B. & Lutcavage M. Dispersal routes and habitat utilization of juvenile Atlantic bluefin tuna, *Thunnus thynnus*, tracked with Mini PSAT and archival tags. Plos One 7, e37829, doi: 10.1371/journal.pone.0037829 (2012). 22629461 PMC3358288

[b79] Core Team R.. R: A language and environment for statistical computing. R Foundation for Statistical Computing, Vienna, Austria. ISBN 3–900051–07–0, URL http://www.R-project.org/ (2012).

[b80] Leben R. R. Altimeter-Derived Loop Current Metrics In Circulation in the Gulf of Mexico: Observations and Models 181–201 (American Geophysical Union, 2005).

[b81] Wood S. N. Generalized Additive Models: An Introduction with R. (Chapman & Hall, 2006).

[b82] Diggle P. J., Heagerty P., Liang K.-Y. & Zeger S. L. Analysis of Longitudinal Data. 2nd edn, (Oxford University Press, 2002).

