# Supplementary material for: Sailfish migrations connect productive coastal areas in the West Atlantic Ocean
Source: Sci Rep. 2016 Dec 1;6:38163. doi: 10.1038/srep38163 (PMC5131301; doi:10.1038/srep38163)
Supplement: Supplementary Information [file srep38163-s1.pdf]

Sailfish migrations connect productive coastal areas in the West Atlantic Ocean

Short title: Sailfish migrations in the Atlantic

Authors

Chi Hin Lam[1,*], Benjamin Galuardi[2], Anthony Mendillo[3], Emily Chandler[1] and Molly E. Lutcavage[1]

Affiliations

[1] Large Pelagics Research Center, School for the Environment, University of Massachusetts Boston, P.O. Box 3188, Gloucester, MA 01931, USA

[2] School of Marine Science and Technology, University of Massachusetts Dartmouth, Fairhaven, MA 02719

[3] Keen M International Fishing Charters, Isla Mujeres, Quintana Roo, Mexico

*Corresponding author: tim.lam@umb.edu; Tel: +1-978-238-8208

Supplementary Materials

Figure S1 Sailfish tracks. Tracks not shown in Figure 1 are displayed. Positions are color-coded by months. Confidence regions at 95% associated with estimated positions are indicated by grey shading. Tag IDs and fish lower jaw fork lengths are noted in the bottom right labels. Tagging locations, green triangle; popoff locations, red triangle; contour of bottom depth at 1000 m, pink line; Loop Current and eddies (annual average), green lines. Refer to Figure S2 for names of geographic and oceanographic features. Maps were generated in R[1] (v2.15.2).

Figure S2 Geographic extent covered by tagged sailfish. Maps were generated in R[1] (v2.15.2). Map and image data: Google, US Dept of State Geographer, Image Landsat, SIO, NOAA, U.S. Navy, NGA, GEBCO.

Figure S3 Conventional tag recoveries for Atlantic sailfish by quarters. Lines connect release and recapture positions, colored by the month of recapture. Recapture positions are indicated by symbols that coded days at liberty (DAL) for an individual into the following categories: <90, 90-179, 180-269, 270-364, and 365-539 days. Recoveries with DAL beyond 540 days are excluded. Maps were generated in R[1] (v2.15.2). Map and image data: Google, US Dept of State Geographer, Image Landsat, SIO, NOAA, U.S. Navy, NGA, GEBCO.

Figure S4 Diagnostic plots for generalized additive mixed model on maximum depth of tagged sailfish. Six plots are available for each model (from left to right): 1) square-root-transformed fitted values versus square-root-transformed observed values, 2) Pearson residuals versus square-root-transformed fitted values, 3) raw residuals versus square-root-transformed fitted values, 4) distribution of normalized residuals, 5) quantile-quantile (Q-Q) plot for Pearson

residuals of the full model, and 6) Q-Q plot for Pearson residuals of the random effect (tagged fish). To aid visualization, a 1:1 line for plots 1, 5 and 6, a zero-centered line for plot 2, and lines of 1 standard deviation (solid) and 2 standard deviation (dotted) for plot 3 are added.

References

1 R Core Team. R: A language and environment for statistical computing. R Foundation for Statistical Computing, Vienna, Austria. ISBN 3-900051-07-0, URL http://www.R-project.org/. (2012).

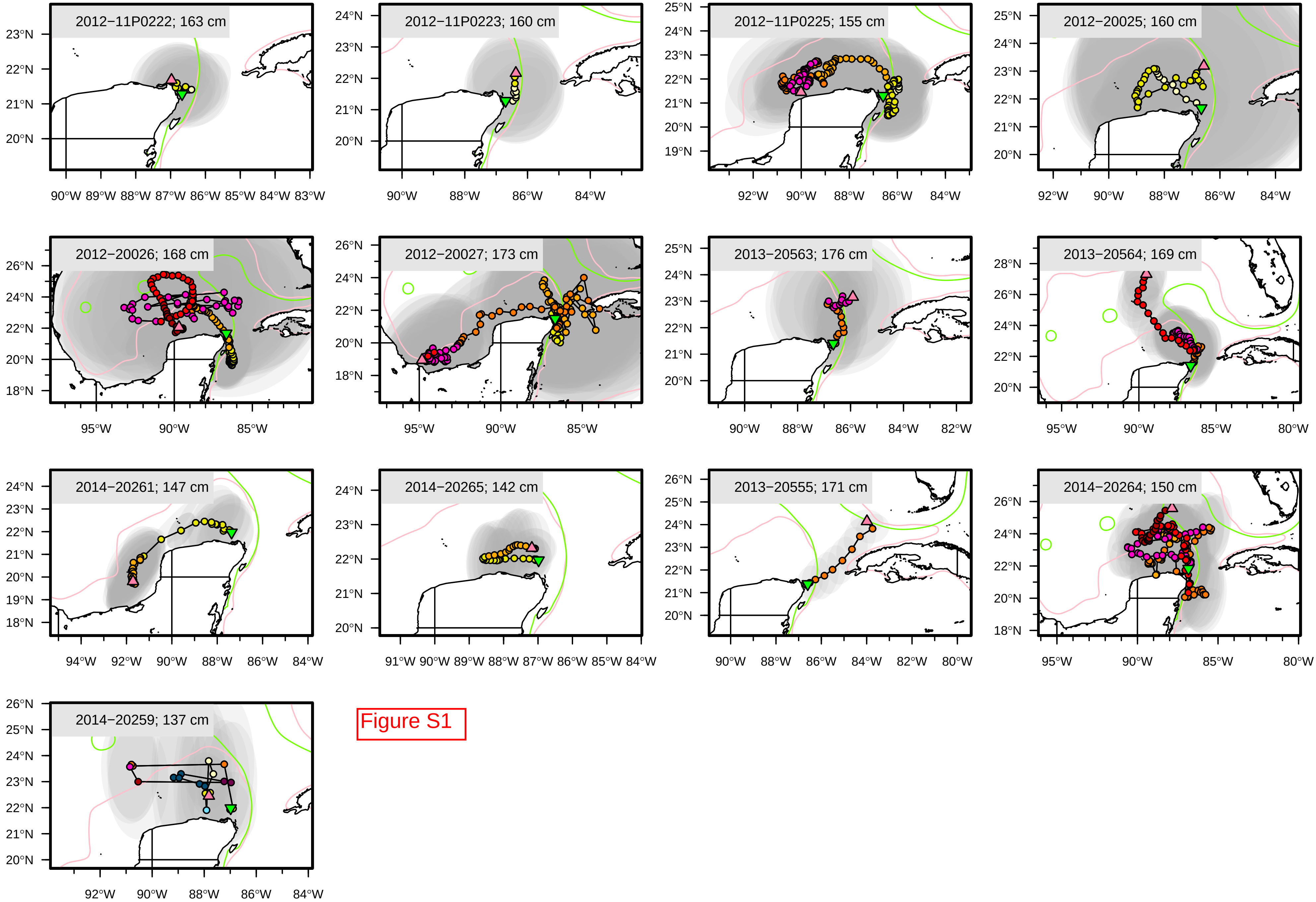
2012−11P0222; 163 cm
2012−11P0223; 160 cm
2012−11P0225; 155 cm
2012−20025; 160 cm
2012−20026; 168 cm
2012−20027; 173 cm
2013−20563; 176 cm
2013−20564; 169 cm
2014−20261; 147 cm
2014−20265; 142 cm
2013−20555; 171 cm
2014−20264; 150 cm
2014−20259; 137 cm

Figure S1

Figure S2

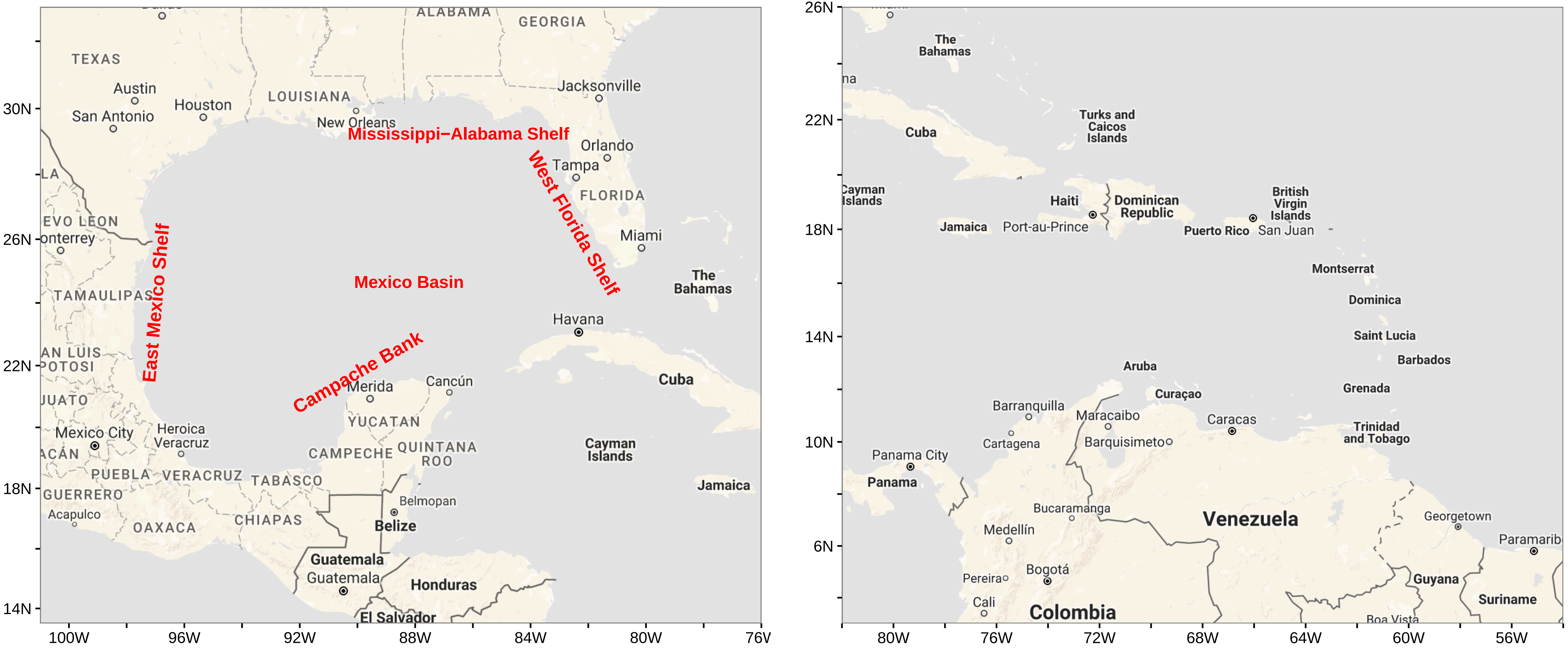
Mississippi–Alabama Shelf
West Florida Shelf
Mexico Basin
East Mexico Shelf
Campache Bank
TEXAS
LOUISIANA
ALABAMA
GEORGIA
FLORIDA
Austin
San Antonio
Houston
New Orleans
Jacksonville
Orlando
Tampa
Miami
The Bahamas
Havana
Cuba
Cayman Islands
Jamaica
Merida
Cancún
YUCATAN
CAMPECHE
QUINTANA ROO
TABASCO
VERACRUZ
PUEBLA
GUERRERO
OAXACA
CHIAPAS
Heroica Veracruz
Mexico City
Acapulco
TAMAULIPAS
Belmopan
Belize
Guatemala
Honduras
El Salvador
30N
26N
22N
18N
14N
100W
96W
92W
88W
84W
80W
76W
Turks and Caicos Islands
Haiti
Dominican Republic
Port-au-Prince
Puerto Rico
San Juan
British Virgin Islands
Montserrat
Dominica
Saint Lucia
Barbados
Grenada
Trinidad and Tobago
Aruba
Curaçao
Barranquilla
Cartagena
Maracaibo
Barquisimeto
Caracas
Panama City
Panama
Bucaramanga
Medellín
Venezuela
Georgetown
Guyana
Suriname
Bogotá
Pereira
Cali
Colombia
10N
6N
72W
68W
64W
60W
56W

Figure S3

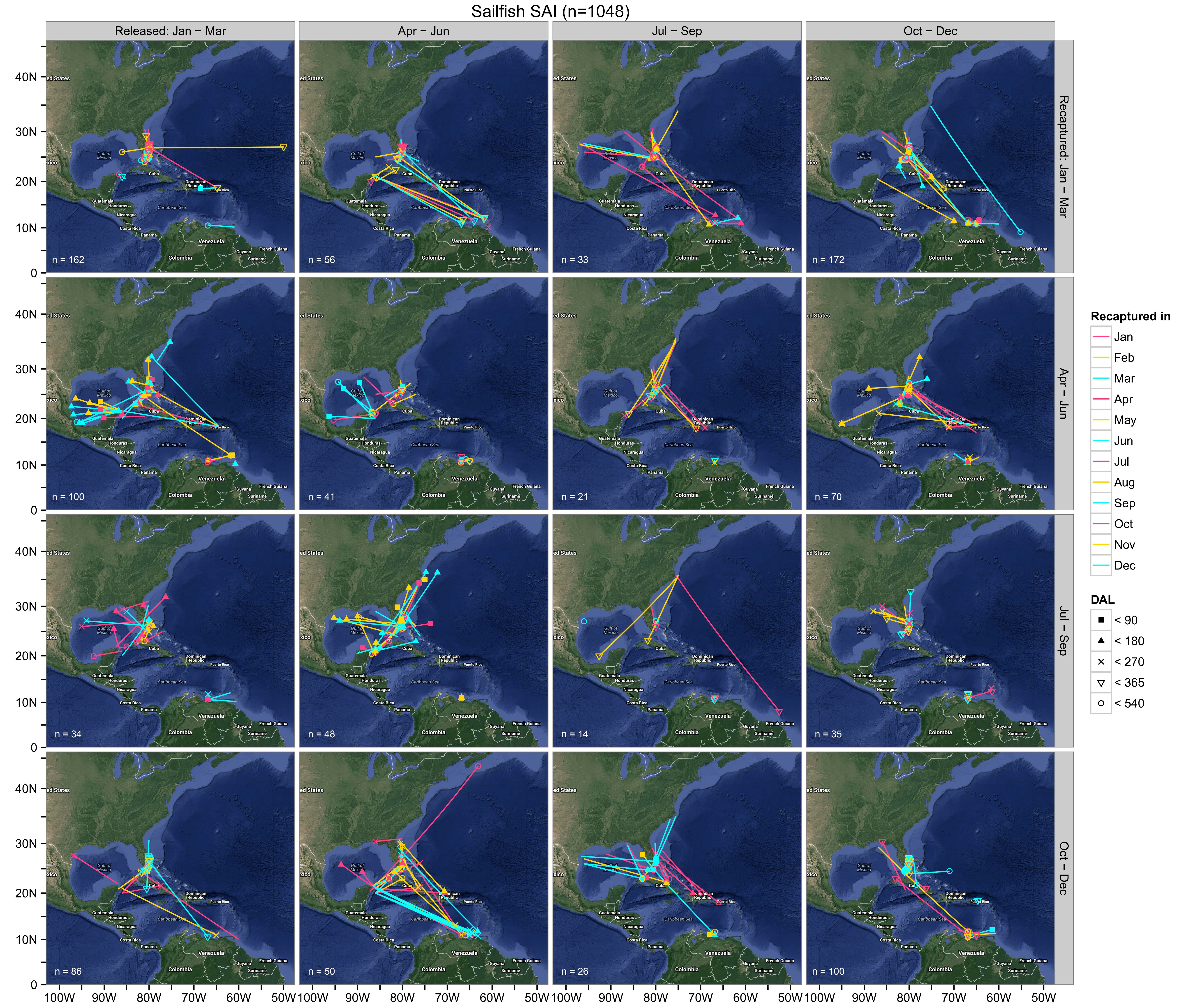

Sailfish SAI (n=1048)
Released: Jan – Mar
Apr – Jun
Jul – Sep
Oct – Dec
Recaptured: Jan – Mar
Apr – Jun
Jul – Sep
Oct – Dec
n = 162
n = 56
n = 33
n = 172
n = 100
n = 41
n = 21
n = 70
n = 34
n = 48
n = 14
n = 35
n = 86
n = 50
n = 26
n = 100
40N
30N
20N
10N
0
100W
90W
80W
70W
60W
50W
Recaptured in
Jan
Feb
Mar
Apr
May
Jun
Jul
Aug
Sep
Oct
Nov
Dec
DAL
< 90
< 180
< 270
< 365
< 540

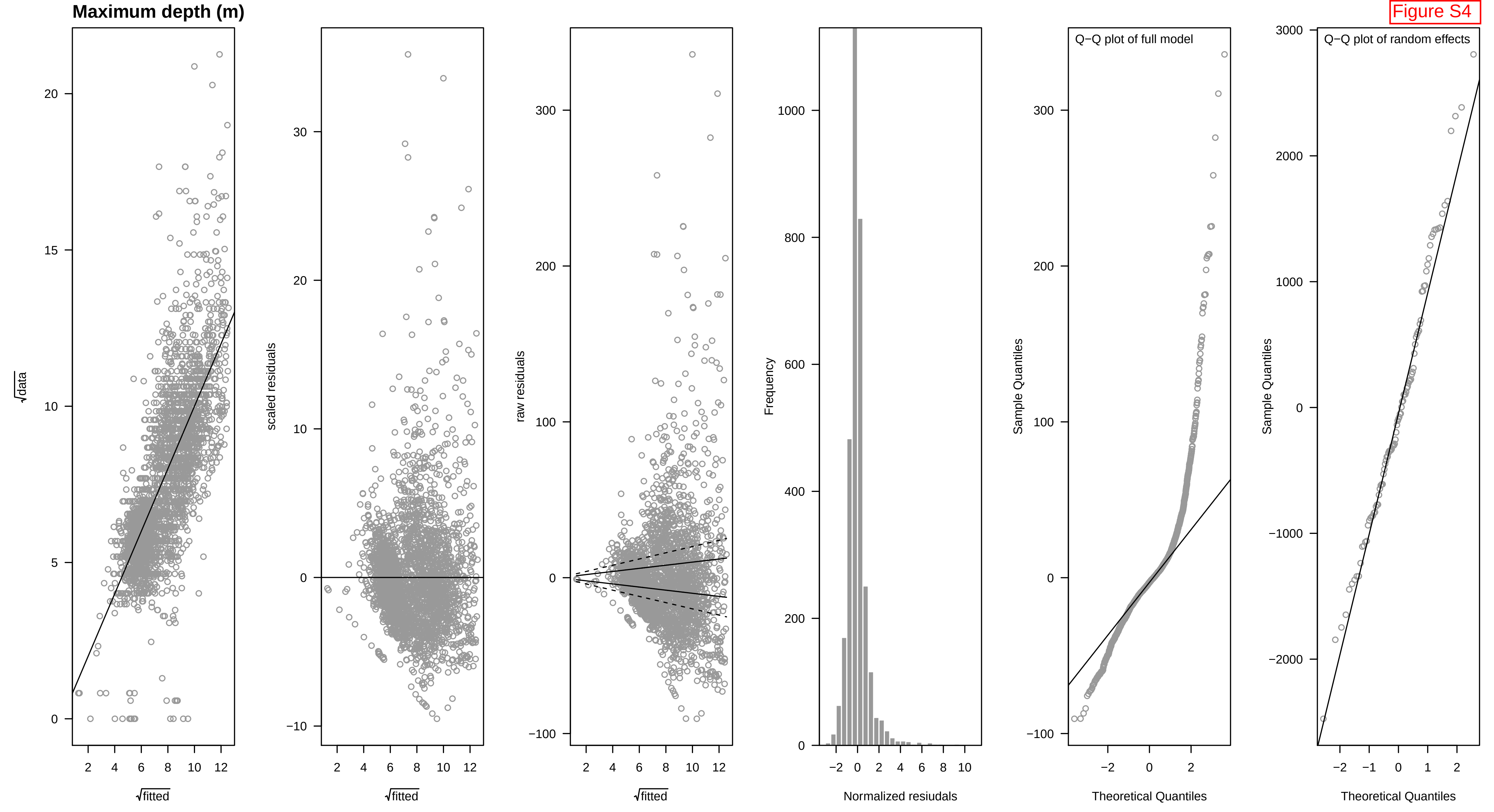

Maximum depth (m)
Figure S4
√data
scaled residuals
raw residuals
Frequency
Sample Quantiles
Q−Q plot of full model
Q−Q plot of random effects
√fitted
Normalized resiuduals
Theoretical Quantiles
